# A new lichenized fungus, *Lecanora baekdudaeganensis*, from South Korea, with a taxonomic key for Korean *Lecanora* species

**DOI:** 10.3897/mycokeys.70.51569

**Published:** 2020-07-24

**Authors:** Beeyoung Gun Lee, Jae-Seoun Hur

**Affiliations:** 1 Baekdudaegan National Arboretum, Bonghwa, 36209, South Korea Baekdudaegan National Arboretum Bonghwa South Korea; 2 Korean Lichen Research Institute, Sunchon National University, Suncheon 57922, South Korea Sunchon National University Suncheon South Korea

**Keywords:** biodiversity, Lecanoraceae, phorophyte, phylogeny, taxonomy

## Abstract

*Lecanora baekdudaeganensis* Lee & Hur is described as a new lichenized fungus from Baekdudaegan Mountains, South Korea. The new species is classified into the *Lecanora subfusca* group – *allophana* type and distinguishable from *Lecanora imshaugii* Brodo by a darker thallus, brownish disc, K–insoluble granules on the surface of the epihymenium, shorter hypothecium, and the presence of oil droplets in the apothecial section. Molecular analyses employing internal transcribed spacer (ITS) and mitochondrial small subunit (mtSSU) sequences strongly support *Lecanora baekdudaeganensis* as a distinct species in the genus *Lecanora*. A surrogate key is provided to assist in the identification of all 52 taxa in the genus *Lecanora* of Korea.

## Introduction

The Baekdudaegan Mountains are the main mountain range stretching across the entire Korean Peninsula. The mountains stretch 1,400 km in length from North Korea to South Korea and encompass protected areas of approximately 2,750 km^2^ (Korea Forest Service 2019). The Baekdudaegan Mountains, as the main mountain system for whole mountainous areas comprising 70 percent of Korea, are almost totally covered with forest and support a productive ecosystem for specialists as well as generalists, represented by 27 percent endemic vascular plants (Korea Forest Service 2019) and 20 percent endemic lichens/lichenicolous fungi.

**Figure 1.**
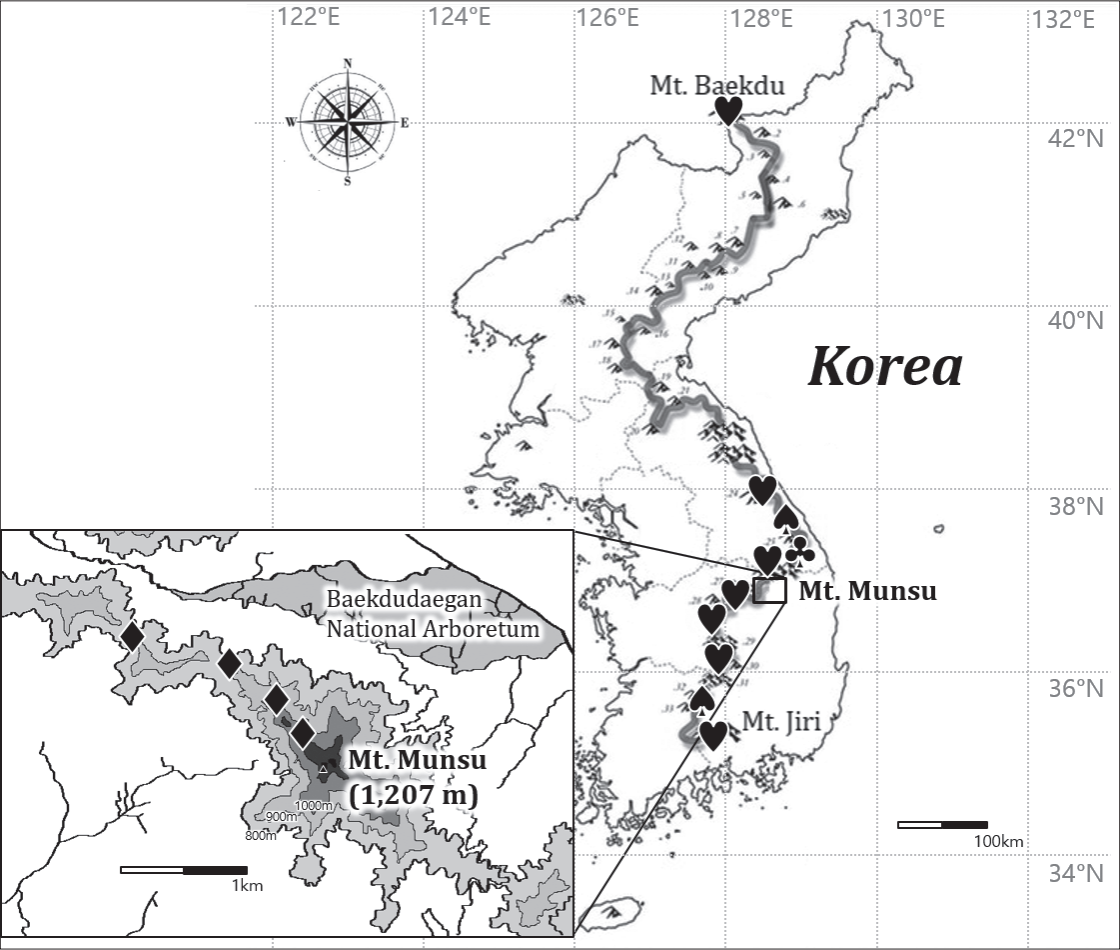
Specific collection sites (black symbols) on the Baekdudaegan Mountains (thick gray line on the entire Korea map), for the new species *Lecanora baekdudaeganensis* (♦), and previously discovered species *L. hafelliana* (♥), *L. loekoesii* (♣), and *L. pseudosambuci* (♠). All *Lecanora* species reported as new species were detected in the Baekdudaegan Mountains or other mountainous areas just close to the mountains in Korea.

Although the genus *Lecanora* is one of the largest genera in lichens, just three new species in *Lecanora* were formerly discovered out of all 164 lichenized or lichenicolous fungi which were reported as new species from Korea. Specifically, all three species, *L. hafelliana* L. Lü, Y. Joshi & Hur, *L. loekoesii* L. Lü, Y. Joshi & Hur, and *L. pseudosambuci* S.Y. Kondr., Lőkös & Hur were detected from the bark of *Quercus* or other deciduous trees in the Baekdudaegan mountains or other mountainous areas in North Korea and South Korea (Lü et al. 2011; Kondratyuk et al. 2016) (Fig. 1).

This study describes a new lichenized fungus in the genus *Lecanora*. During three field trips to Mt. Munsu, Bonghwa in 2019 (Fig. 1), four specimens were collected but identified just to genus without matching any previously known species. We describe them below as a new corticolous lichen species, *Lecanora baekdudaeganensis*, and this discovery contributes to the taxonomy with overall 52 taxa in the genus *Lecanora* from North Korea and South Korea. All specimens are deposited in the herbarium of the Baekdudaegan National Arboretum (BDNA), South Korea.

## Materials and methods

### Morphological and chemical analyses

Hand-cut sections were prepared with a razor blade under a stereomicroscope (Olympus optical SZ51; Olympus, Tokyo, Japan), examined under a compound microscope (Nikon Eclipse E400; Nikon, Tokyo, Japan) and imaged using a software program (AxioVision Release 4.8.2; Carl Zeiss, Jena, Germany) and an Axiocam ERc 5s camera (Carl Zeiss, Jena, Germany) mounted on a Zeiss Axioscope A1 microscope (Carl Zeiss, Jena, Germany). The ascospores were investigated at 1000× magnification in water. The length and width of the ascospores were measured and the range of spore sizes was shown with average, standard deviation, and number of measured spores. Thin-layer chromatography (TLC) was performed using solvent systems A and C according to standard methods (Orange et al. 2001).

### Isolation, DNA extraction, amplification, and sequencing

Hand-cut sections of ascomata or thallus from all collected specimens were prepared for DNA isolation and DNA was extracted with a NucleoSpin Plant II Kit in line with the manufacturer’s instructions (Macherey-Nagel, Düren, Germany). PCR amplification for the internal transcribed spacer region (ITS1-5.8S-ITS2 rDNA) and the mitochondrial small subunit genes was achieved using Bioneer’s AccuPower PCR Premix (Bioneer, Daejeon, Korea) in 20-μL tubes and primers ITS5 and ITS4 (White et al. 1990), and mrSSU1 and mrSSU3R (Zoller et al. 1999), respectively. The PCR thermal cycling parameters used were 95 °C (15 sec), followed by 35 cycles of 95 °C (45 sec), 54 °C (45 sec), and 72 °C (1 min), and a final extension at 72 °C (7 min) based on Ekman (2001). DNA sequences were generated by the genomic research company GenoTech (Daejeon, Korea).

### Phylogenetic analyses

All ITS and mtSSU sequences were aligned and edited manually using ClustalW in Bioedit V7.2.6.1 (Hall 1999). All missing and ambiguously aligned data and parsimony-uninformative positions were removed and only parsimony-informative regions were finally analyzed in MEGA X (Stecher et al. 2020). The final alignment comprised 564 (ITS) and 1032 (mtSSU) columns. In them, variable regions were 51 (ITS) and 100 (mtSSU). Finally, the phylogenetically informative regions were 359 (ITS) and 464 (mtSSU). Phylogenetic trees with bootstrap values were obtained in RAxML GUI 2.0 beta (Edler et al. 2019) using the maximum likelihood method with a rapid bootstrap with 1000 bootstrap replications and GTR GAMMA for the substitution matrix. The posterior probabilities were obtained in BEAUti 1.8.0 and BEAST 1.8.0 (Drummond et al. 2012) using the HKY (Hasegawa, Kishino and Yano) model, as the appropriate model of nucleotide substitution based on the Bayesian Information Criterion (BIC) (Schwarz 1978) as evaluated by bModelTest (Bouchaert and Drummond 2017), empirical base frequencies, gamma for the site heterogeneity model, four categories for gamma, and a 10,000,000 Markov chain Monte Carlo chain length with a 10,000-echo state screening and 1000 log parameters. Then, a consensus tree was constructed in TreeAnnotator 1.8.0 (Drummond and Rambaut 2007) with a burn-in of 5000, no posterior probability limit, a maximum clade credibility tree for the target tree type, and median node heights. All trees were displayed in FigTree 1.4.2 (Rambaut 2014) and edited in Microsoft Paint. The bootstrapping and Bayesian analyses were repeated three times for the result consistency and no significant differences were shown for the tree shapes and branch values. The phylogenetic trees and DNA sequence alignments are deposited in TreeBASE under the study ID 25859.

## Results and discussion

### Phylogenetic analyses

**Figure 2.**
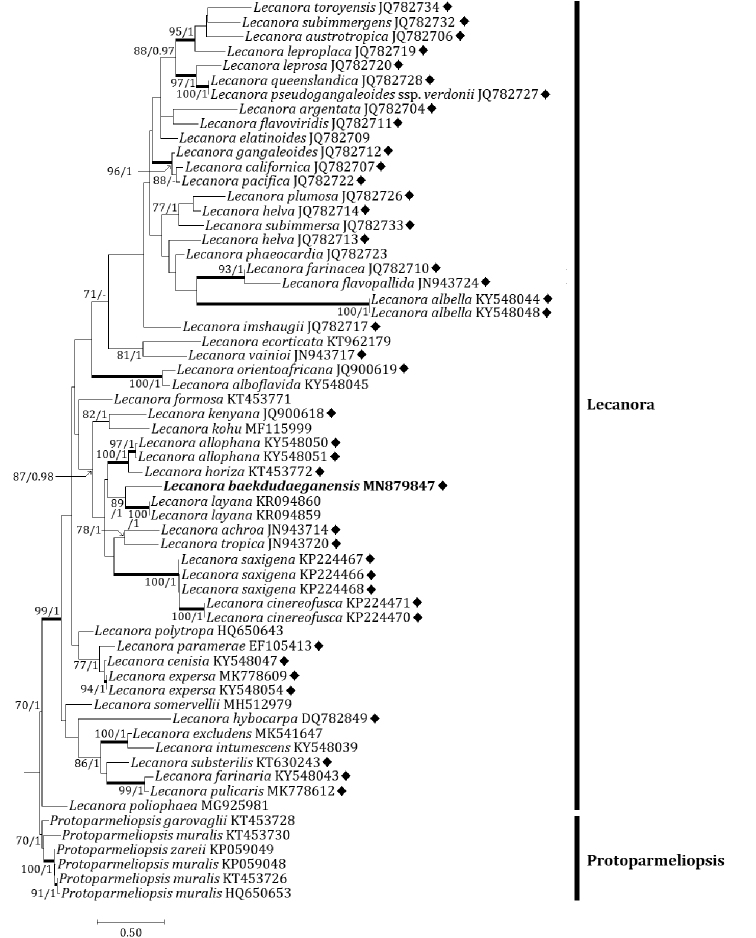
Phylogenetic relationships among comparable species related mainly with the *Lecanora subfusca* group based on a maximum likelihood analysis of the nuclear ribosomal ITS1-5.8S-ITS2 region. The tree was rooted with several sequences in the genus *Protoparmeliopsis*. Maximum likelihood bootstrap values ≥ 70% and posterior probabilities ≥ 95% are shown above internal branches. Branches with bootstrap values ≥ 90% are shown in bold. The new species *Lecanora baekdudaeganensis* is presented in bold, and all species names are followed by GenBank accession numbers. A dash indicates branches with posterior probabilities <95%. The *Lecanora subfusca* group is marked with a black diamond (♦). Reference Table 1 provides the GenBank accession numbers for the included species and voucher information.

**Figure 3.**
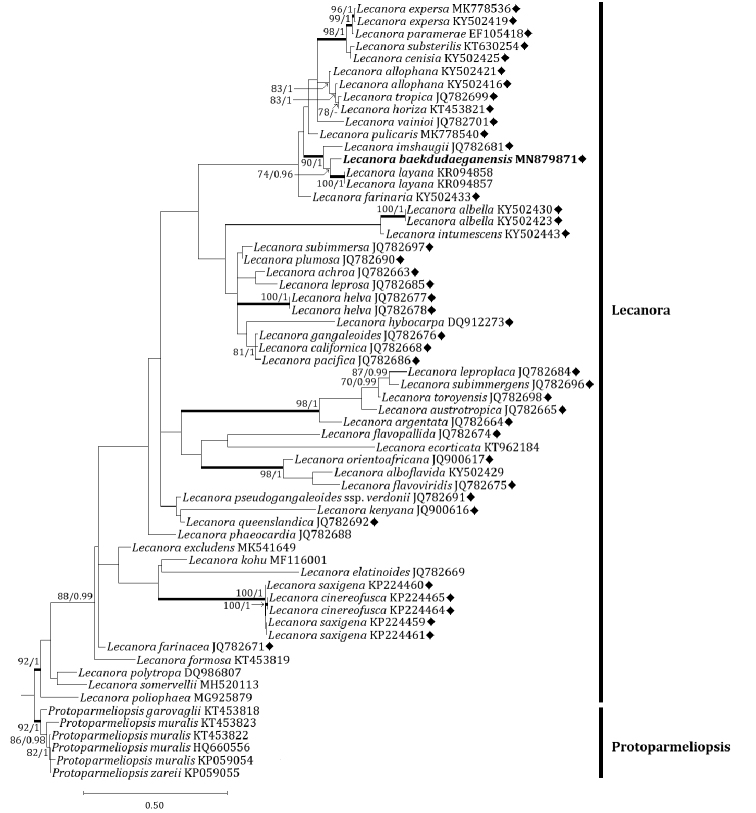
Phylogenetic relationships among comparable species related mainly with the *Lecanora subfusca* group based on a maximum likelihood analysis of the mitochondrial small subunit (mtSSU) sequences. The tree was rooted with several sequences in the genus *Protoparmeliopsis*. Maximum likelihood bootstrap values ≥ 70% and posterior probabilities ≥ 95% are shown above internal branches. Branches with bootstrap values ≥ 90% are shown in bold. The new species *Lecanora baekdudaeganensis* is presented in bold, and all species names are followed by GenBank accession numbers. A dash indicates branches with posterior probabilities <95%. The *Lecanora subfusca* group is marked with a black diamond (♦). Reference Table 1 provides the GenBank accession numbers for the included species and voucher information

Two independent phylogenetic trees for the *Lecanora subfusca* group and related species were produced from 122 sequences (61 for ITS, and 61 for mtSSU) from GenBank and with two new sequences (each one for ITS and mtSSU) for the new species (Table 1). The new species was positioned in the *L. subfusca* group in both ITS and mtSSU trees. In the ITS tree, the new species was located in a clade with *L. achroa* Nyl., *L. allophana* (Ach.) Nyl., *L. cinereofusca* H. Magn., *L. horiza* (Ach.) Röhl., *L. layana* Lendemer, *L. saxigena* Lendemer & R.C. Harris and *L. tropica* Zahlbr. (Fig. 2). All species including the new species were in the *L. subfusca* group except for *L. layana* which was nevertheless the most closely located to the new species, represented by a bootstrap value of 89 and a posterior probability of 100 for the branch. Many other species, including *L. imshaugii* in the *L. subfusca* group, were positioned in different clades and our results did not reveal any close species in the *L. subfusca* group to the new species. In the mtSSU tree, the new species is located in a clade with *L. allophana*, *L. cenisia* Ach., *L. expersa* Nyl., *L. farinaria* Borrer, *L. horiza*, *L. imshaugii*, *L. layana*, *L. paramerae* I. Martínez, Aragón & Lumbsch, *L. pulicaris* (Pers.) Ach., *L. substerilis* Malíček & Vondrák, *L. tropica*, and *L. vainioi* Vänskä (Fig. 3). All species including the new species were in the *L. subfusca* group except for *L. layana*. Except for *L. layana*, a sorediate species (Lendemer 2015), *L. imshaugii* was the most closely positioned with the new species, represented by a bootstrap value of 90 and a posterior probability of 100 for the branch. Our analysis did not represent any species identical to the new species in the *L. subfusca* group.

**Table 1.** Species list and DNA sequence information employed for phylogenetic analysis.

No.	Species	ID (ITS)	ID (mtSSU)	Voucher
1	*Lecanora achroa*	JN943714	JQ782663	Papong 6458
2	*Lecanora albella*	KY548044	KY502430	Berger 29362
3	*Lecanora albella*	KY548048	KY502423	Malicek 7336
4	*Lecanora alboflavida*	KY548045	KY502429	Coppins s.n.
5	*Lecanora allophana*	KY548050	KY502421	Malicek 9626
6	*Lecanora allophana*	KY548051	KY502416	Malicek 9491
7	*Lecanora argentata*	JQ782704	JQ782664	Papong 6041(F)
8	*Lecanora austrotropica*	JQ782706	JQ782665	Papong 6407(F)
**9**	***Lecanora baekdudaeganensis***	**MN879847**	**MN879871**	**BDNA-L-0000065**
10	*Lecanora californica*	JQ782707	JQ782668	Lumbsch 19914a(F)
11	*Lecanora cenisia*	KY548047	KY502425	Malicek 5869
12	*Lecanora cinereofusca*	KP224470	KP224465	Lendemer 34944 (NY)
13	*Lecanora cinereofusca*	KP224471	KP224464	Lendemer 35007 (NY)
14	*Lecanora ecorticata*	KT962179	KT962184	NMW<GBR>:C.2015.005.77
15	*Lecanora elatinoides*	JQ782709	JQ782669	Lumbsch 19992d(F)
16	*Lecanora excludens*	MK541647	MK541649	Palice 21929
17	*Lecanora expersa*	KY548054	KY502419	Malicek 9625
18	*Lecanora expersa*	MK778609	MK778536	Vondrak 16033 (PRA)
19	*Lecanora farinacea*	JQ782710	JQ782671	Lumbsch 20022d(F)
20	*Lecanora farinaria*	KY548043	KY502433	Tonsberg 46170
21	*Lecanora flavopallida*	JN943724	JQ782674	Lumbsch 20031a
22	*Lecanora flavoviridis*	JQ782711	JQ782675	Papong 6539(F)
23	*Lecanora formosa*	KT453771	KT453819	ZX 20129045-2
24	*Lecanora gangaleoides*	JQ782712	JQ782676	Lumbsch 19923a(F)
25	*Lecanora helva*	JQ782713	JQ782677	Lumbsch 19809h(F)
26	*Lecanora helva*	JQ782714	JQ782678	Lumbsch 19843b(F)
27	*Lecanora horiza*	KT453772	KT453821	Zhao 2015
28	*Lecanora hybocarpa*	DQ782849	DQ912273	AFTOL-ID 639
29	*Lecanora imshaugii*	JQ782717	JQ782681	Lumbsch 19273b(F)
30	*Lecanora intumescens*	KY548039	KY502443	Malicek 8203
31	*Lecanora kenyana*	JQ900618	JQ900616	Kirika 1179 (F)
32	*Lecanora kohu*	MF115999	MF116001	UNITEC 7497
33	*Lecanora layana*	KR094859	KR094857	Lendemer 37519 (NY)
34	*Lecanora layana*	KR094860	KR094858	Lendemer 38131 (NY)
35	*Lecanora leproplaca*	JQ782719	JQ782684	Lumbsch 19815r(F)
36	*Lecanora leprosa*	JQ782720	JQ782685	Papong 6443(F)
37	*Lecanora orientoafricana*	JQ900619	JQ900617	Kirika 2205(F)
38	*Lecanora pacifica*	JQ782722	JQ782686	Lumbsch 19901c(F)
39	*Lecanora paramerae*	EF105413	EF105418	Lumbsch s.n. (F)
40	*Lecanora phaeocardia*	JQ782723	JQ782688	Papong 3473(F)
41	*Lecanora plumosa*	JQ782726	JQ782690	Papong 6965(F)
42	*Lecanora poliophaea*	MG925981	MG925879	O:L 200460
43	*Lecanora polytropa*	HQ650643	DQ986807	AFTOL-ID 1798
44	*Lecanora pseudogangaleoides* subsp. verdonii	JQ782727	JQ782691	Lumbsch 19103a(F)
45	*Lecanora pulicaris*	MK778612	MK778540	Malicek 10263
46	*Lecanora queenslandica*	JQ782728	JQ782692	Lumbsch 19113j(F)
47	*Lecanora saxigena*	KP224466	KP224459	Lendemer 32825 (NY)
48	*Lecanora saxigena*	KP224467	KP224460	Lendemer 25832 (NY)
49	*Lecanora saxigena*	KP224468	KP224461	Lendemer 33186 (NY)
50	*Lecanora somervellii*	MH512979	MH520113	YO 10109
51	*Lecanora subimmergens*	JQ782732	JQ782696	Papong 6431(F)
52	*Lecanora subimmersa*	JQ782733	JQ782697	Lumbsch 19103b(F)
53	*Lecanora substerilis*	KT630243	KT630254	Malicek 202
54	*Lecanora toroyensis*	JQ782734	JQ782698	Papong 7197(F)
55	*Lecanora tropica*	JN943720	JQ782699	Papong 6440
56	*Lecanora vainioi*	JN943717	JQ782701	Papong 6957
57	*Protoparmeliopsis garovaglii*	KT453728	KT453818	Leavitt 089 (BRY-C)
58	*Protoparmeliopsis muralis*	HQ650653	HQ660556	Schmull s. n.
59	*Protoparmeliopsis muralis*	KP059048	KP059054	SK 765
60	*Protoparmeliopsis muralis*	KT453726	KT453822	Leavitt 143 (BRY-C)
61	*Protoparmeliopsis muralis*	KT453730	KT453823	Vondrak 9413
62	*Protoparmeliopsis zareii*	KP059049	KP059055	SK 480
	**Overall**	**62**	**62**	

DNA sequences for the new species *Lecanora baekdudaeganensis* (in bold) were generated in this study. All others were obtained from GenBank. The species names are followed by GenBank accession numbers and voucher information. ITS, internal transcribed spacer; mtSSU, mitochondrial small subunit; Voucher, voucher information.

### Taxonomy

#### Lecanora baekdudaeganensis

Taxon classificationFungiLecanoralesLecanoraceae

B.G. Lee & J-.S. Hur sp. nov.

96470313-35EC-5A83-BB6F-B5B4B1D97771

833845

Fig. 4

##### Diagnosis.

*Lecanora baekdudaeganensis* differs from *L. imshaugii* by a darker thallus (bluish, olivish, or pale brownish gray vs. greenish or yellowish gray), brownish disc (vs. reddish brown disc), K-insoluble granules on the surface of epihymenium (vs. absence of granules), shorter hypothecium (15–25 μm vs. 50–75 μm), and the presence of oil droplets in the apothecial section.

**Figure 4.**
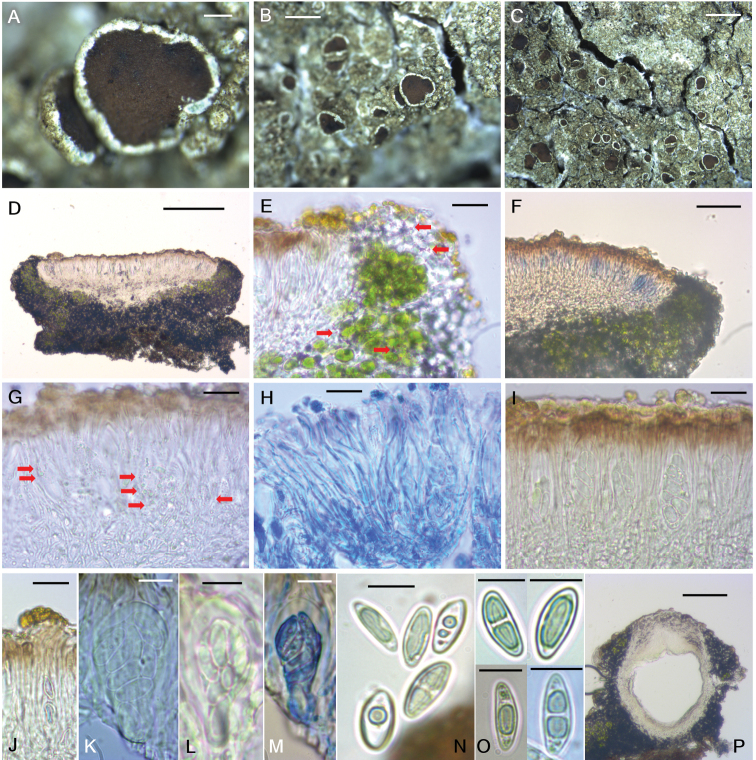
*Lecanora baekdudaeganensis* (BDNA-L-0000065, holotype) in morphology. **A–C** Habitus with dark thallus and epruinose apothecia **D** sessile apothecia with constricted base in section. Hypothecial base closed by medulla of amphithecium **E** well-developed amphithecium with small calcium oxalate crystals (red arrows) not dissolving in KOH **F** apothecial section in Iodine. I– reaction in the beginning then turning slowly to blue or purple–blue hymenium **G** oil droplets (red arrows) present in the apothecial section **H** anastomosing paraphyses shown in Lactophenol cotton blue **I** asci in hymenium **J** epihymenium with yellowish granules not dissolving in KOH **K–M** 8-spored, clavate asci (**M** in Lactophenol cotton blue) **N, O** ellipsoid ascospores in diverse development stages. Spores biguttulate in the beginning then having a long oil drop by assembly of guttules when mature **P** old pycnidia without pycnocodidia. Scale bars: 200 μm (**A, D**), 2 mm (**B, C**), 20 μm (**E, G–J**), 50 μm (**F**), 10 μm (**K–O**), 100 μm (**P**).

##### Type.

South Korea, North Gyeongsang Province, Bonghwa-gun, Chunyang-myeon, Mt. Munsu, 36°59.41'N, 128°48.24'E, 1,005 m alt., on bark of *Quercus mongolica* Fisch. ex Ledeb., 29 August 2019, B.G.Lee 2019-000065 (holotype: BDNA-L-0000065!; GenBank MN879847 for ITS and MN879871 for mtSSU); South Korea, North Gyeongsang Province, Bonghwa-gun, Chunyang-myeon, Mt. Munsu, 37°0.31'N, 128°47.39'E, ca 900 m alt., on bark of *Quercus dentata* Thunb., 26 September 2019, B.G.Lee 2019-000135 (paratype: BDNA-L-0000135); South Korea, North Gyeongsang Province, Bonghwa-gun, Chunyang-myeon, Mt. Munsu, 36°59.82'N, 128°46.81'E, 970 m alt., on bark of *Quercus mongolica*, 26 August 2019, B.G.Lee 2019-000147 (paratype: BDNA-L-0000147); South Korea, North Gyeongsang Province, Bonghwa-gun, Chunyang-myeon, Mt. Munsu, 36°59.35'N, 128°46.12'E, ca 1,075 m alt., on bark of *Quercus mongolica*, 26 August 2019, B.G.Lee 2019-000151 (paratype: BDNA-L-0000151).

##### Description.

Thallus corticolous, crustose, without lobes, continuous or cracked, rimose to areolate or verruculose, usually rounded or irregular, bluish gray in the beginning (margin) and olivish– or pale brownish–gray when mature (center), not pruinose, 30–70 mm diam., 100–170 μm thick; cortex hyaline to pale yellow or pale brown, 5–10 μm thick; medulla 20–75 μm thick; photobiont coccoid, forming a distinct algal layer, 45–80 μm thick, cells globose, 8.5–17 × 8–15 μm. Prothallus absent.

Apothecia abundant, rounded, smaller and scattered around the margin and larger and aggregated in the center, constricted at the base, 0.2–1.6 mm diam. Disc flat to slightly concave, not pruinose, brown to dark brown from the beginning, 270–430 μm thick; margin persistent, prominent, generally entire or slightly flexuous, some a little crenulate when old, concolorous to thallus. Amphithecium well-developed, with numerous small crystals in both algal-containing and cortical parts (*allophana*-type) not dissolving in K, 60–100 μm thick laterally, 110–130 μm thick basally; amphithecial cortex distinct, 7–12 μm thick. Parathecium hyaline, indistinct in water, 15–25 μm thick in I. Epihymenium pale yellowish brown to pale brown, with small granules on the surface not dissolving in K, pigment slightly paler in K but not diluted, without oil droplets, 5–15 μm high. Hymenium hyaline, 50–75 μm high. Subhymenium hyaline, 20–40 μm high. Hypothecium hyaline, coarsely prosoplectenchymatous (periclinal) in the lower and marginal parts and prosoplectenchymatous (irregular) in the upper and central parts, 15–25 μm high. Oil droplets present in hypothecium, subhymenium and the base of hymenium. Hypothecial base not extending or a little extending to the substrate and always closed by medulla of amphithecium. Paraphyses septate, anastomosing, 1–2.5 μm wide, simple or sparsely branched at tips but not, or only slightly, swollen, 2.5–4 μm. Asci clavate, 8-spored, 41–51 × 13–20 μm (n = 10). Ascospores simple, often biguttulate in the beginning then having an oval-shaped oil drop by assembly of guttules when mature, narrowly or widely ellipsoid, or eye-shaped, 10–18.5 × 4.5–9.5 μm (mean = 15.2 × 6.5; SD = 1.58 (L), 1.10 (W); n = 128), wall ca 0.5 μm thick when exist. Pycnidia only once detected, pale brown at tip, ovoid, 315 × 330 μm, without conidia as old.

##### Chemistry.

Thallus K+ yellow, KC+ yellow, C–, Pd–. Hymenium I– in the beginning but turning slowly blue or purple–blue, KI+ blue (reaction mainly starting from tholus then the whole ascus), C–, Pd–. UV–. Atranorin, zeorin and an unidentified minor constituent (Rf classes A3 and C3 in Culberson’s standardized thin layer chromatography method (Culberson 1972)), UV– before heating, spot color slightly pale yellow-orange after heating, and UV+ pale pink-orange after heating) were detected by TLC.

##### Distribution and ecology.

The species occurs on the bark of *Quercus mongolica* and *Q. dentata* which are the most dominant tree species on the mountain. This species is currently known from four different sites on the mountain.

##### Etymology.

The species epithet indicates the lichen’s geography, namely the main mountains called Baekdudaegan stretching from north to south in the entire Korean Peninsula.

##### Notes.

The new species is classified to the *Lecanora subfusca* group – *allophana* type, representing the main characteristics of a crustose thallus without lobes containing atranorin as a major constituent, small calcium oxalate crystals in both algal-containing and cortical parts of the amphithecium, and trebouxioid photobionts in the thalline margin, dark brown discs, and colorless ellipsoid simple spores in the range of 10–20 × 6–9 μm (Brodo 1984; Miyawaki 1988; Lumbsch 1995; Lumbsch et al. 2003). The new species is compared with *Lecanora chionocarpa* Hue, *L. horiza*, *L. imshaugii*, *L. japonica* Müll. Arg., and *L. megalocheila* (Hue) H. Miyaw., as those species are in the *L. subfusca* group with only small crystals in the amphithecium (*allophana or campestris* type) which is defined by the main characteristics such as K+ yellow thallus reaction (containing atranorin), small calcium oxalate crystals in algal-containing and/or cortical parts of amphithecium, and ascospores in the size of 10–20 × 6–9 μm (Hue 1915; Brodo 1984; Miyawaki 1988; Smith et al. 2009). The new species is most similar to *L. imshaugii* by a continuous, rimose, verruculose or areolate thallus, the absence of soredia, the absence of a prothallus, apothecia size, and ascospore size (Brodo 1984). However, *Lecanora baekdudaeganensis* differs from *L. imshaugii* by a darker thallus (bluish, olivish, or pale brownish gray vs. greenish or yellowish gray), brownish disc (vs. reddish brown disc), K–insoluble granules on the surface of epihymenium (vs. absence of granules), a shorter hypothecium (15–25 μm vs. 50–75 μm), and the presence of oil droplets in the apothecial section (Brodo 1984).

The new species is distinguishable from *L. chionocarpa* by a darker thallus (bluish, olivish or pale brownish gray vs. ash gray), the absence of a prothallus (vs. presence of white prothallus), crystals in the amphithecium not dissolving in K (vs. granular crystals dissolving in K), a shorter hymenium (50–75 μm vs. 75–100 μm), the presence of oil droplets in hypothecium, subhymenium and the base of hymenium (vs. oil droplets present in epihymenium), shorter asci (41–51 × 13–20 μm vs. 60–70 × 13–18 μm), smaller ascospores (10–18.5 × 4.5–9.5 μm vs. 15–20 × 8–11 μm), thinner ascospore walls (0.5 μm vs. 0.5–1 μm), and the Pd– reaction of the thallus and medulla (vs. Pd+ yellowish) (Hue 1915; Miyawaki 1988).

The new species differs from *L. horiza* by a darker thallus (bluish, olivish or pale brownish gray vs. yellowish white to whitish gray), and the crystals in amphithecium not dissolving in K (vs. crystals dissolving in K) (Smith et al. 2009).

The new species differs from *L. japonica* by thallus color (bluish, olivish or pale brownish gray vs. dirty greenish to ashy gray), the absence of a prothallus (vs. prothallus with white bundle of hyphae), larger apothecia (0.2–1.6 mm vs. up to 1 mm), a thicker amphithecium (60–100 μm laterally and 110–130 μm basally vs. 5–20 μm laterally and 20–50 μm basally), the presence of oil droplets in hypothecium, subhymenium and the base of hymenium (vs. oil droplets present in epihymenium), shorter hymenium (50–75 μm vs. 70–80 μm), a shorter subhymenium (20–40 μm vs. 180–220 μm), a granular epihymenium (vs. non-granular epihymenium), shorter (41–51 μm) and constantly 8-spored asci (vs. longer (50–80 μm) and 8- or 16-spored asci), the Pd– reaction of the thallus and medulla (vs. Pd+ pale brown thallus and medulla), and the absence of chloroatranorin (vs. presence of chloroatranorin) (Nylander 1891; Miyawaki 1988; Guderley and Lumbsch 1999).

The new species differs from *L. megalocheila* by a darker thallus (bluish, olivish or pale brownish gray vs. whitish gray or whitish with green tinge without brownish color), the absence of a prothallus (vs. blackish prothallus), crystals in the amphithecium not dissolving in K (vs. crystals dissolving in K), a shallower hypothecium (15–25 μm vs. 120–150 μm), wider asci (41–51 × 13–20 μm vs. 35–50 × 10–14 μm), larger ascospores (10–18.5 × 4.5–9.5 μm vs. 10–14 × 5–8 μm), , and the Pd– reaction of the thallus and medulla (vs. Pd+ pale yellow thallus and medulla) (Hue 1915; Miyawaki 1988).

### Key to the species in *Lecanora* of Korea (52 taxa)

Overall, 56 species have been recorded in the genus *Lecanora* from Korea (i.e., South Korea (55 spp.) and North Korea (6 spp.) with sharing five species from both countries). However, four of these species are excluded in the key. *Lecanora fusanii* Hue is regarded as a *Caloplaca* species because *L. fusanii* (syn. *Caloplaca fusanii* (Hue) Zahlbr.) has yellow thalli, orange discs, and polarilocular ascospores (Hue 1915). *Lecanora subrugosa* Nyl. is identical to *L. argentata* (Ach.) Röhl. based on a molecular analysis (Malíček 2014). *Lecanora vulnerata* Hue (syn. *Caloplaca vulnerata* (Hue) Zahlbr.) is supposed to be classified into the family Teloschistaceae because *L. vulnerata* was compared with *L. heppiana* (Müll. Arg.) Hue as a quite similar species, and the former differs from the latter mainly by presence of soredia and KOH reaction (Hue 1915). The latter is classified in the family Teloschistaceae as a *Variospora* species at present (Arup et al. 2013). *Lecanora muralis* (Schreb.) Rabenh., a lobed species, is excluded from the key as it is classified into the genus *Protoparmeliopsis*. However, one species, *L. confusa* Almb., is included in the list as the species was discovered in North Korea. A further five species from North Korea, i.e., *L. chionocarpa*, *L. megalocheila*, *L. polytropa* (Ehrh.) Rabenh., *L. rubina* (Hoffm.) Ach., and *L. subrubra* Hue (syn. *L. japonica*), were previously discovered in South Korea as well.

**Table:** 

1	Thallus saxicolous or lignicolous	**2**
–	Thallus corticolous	**31**
2	Thallus lobate or sublobate	**3**
–	Thallus not lobed	**5**
3	Disc dark, ruby-colored	***L. rubina***
–	Disc light-colored, pale pink, greenish brown to yellow–brown	**4**
4	Thallus usually areolate or sometimes sublobate, paraphyses tips swollen up to 3 μm wide, conidia 20–25 × 1 μm, thallus Pd+ orange	***L. albescens* (*Myriolecis albescens*)**
–	Thallus lobate, resetting, paraphyses tips hardly swollen, conidia absent, thallus Pd–	***L. valesiaca***
5	Thallus inconspicuous, immersed or with dispersed areoles, ascospores 10–14 × 5–6.5 μm, thallus UV–	***L. polytropa***
–	Thallus clearly visible	**6**
6	On calcareous rocks or wood/logs	**7**
–	On non-calcareous rocks	**9**
7	Common on wood/logs, thalline margin excluded finally	***L. anopta***
–	Only on calcareous rocks, thalline margin persistent	**8**
8	Thallus starkly white or pale gray, apothecia 0.1–0.7 mm diam., thallus Pd+ orange	***L. albescens* (*Myriolecis albescens*)**
–	Thallus gray to blackened, apothecia 0.5–1.4 mm diam., thallus Pd–	***L. semipallida* (*Myriolecis semipallida*)**
9	Disc pruinose	**10**
–	Disc not pruinose	**13**
10	Prothallus green–black, thalline margin ±excluded, disc densely gray pruinose, epihymenium green– or blue–gray, containing zeorin, ±gangaleoidin, and usnic acid	***L. sulphurea***
–	Prothallus whitish, thalline margin persistent, containing small or large crystals, disc slightly or faintly pruinose, epihymenium brownish, containing ±chloratranorin	**11**
11	Thalline margin with small, irregular crystals (<10 μm diam.) not dissolving in K, thallus white to yellow–white, K+ indistinct yellow	***L. horiza***
–	Thalline margin with large crystals (>10 μm diam.), thallus grayish, yellowish or brownish, K+ yellow or yellow turning to red	**12**
12	Thallus not glossy, apothecia 1–2 mm diam., disc yellow–brown, red–brown to black, ascospores 9–15 × 6–8.5 μm, containing ±roccellic acid and norstictic acid	***L. cenisia***
–	Thallus somewhat glossy, apothecia 0.4–0.7 mm diam., disc waxy or pale to greenish orange, ascospores 8–11.5 × 4–6.5 μm	***L. plumosa***
13	Disc blackish, epihymenium bluish or greenish, not reddish, orangish or brownish	**14**
–	Disc yellowish to brownish, epihymenium yellowish to brownish	**16**
14	Thallus grayish white to white, prothallus blackish when present, ascospores smaller, 7–13 × 4–6.5 μm, thallus Pd+ yellow, medulla Pd+ pale yellow	***L. oreinoides***
–	Thallus green–yellow to yellowish, prothallus absent, ascospores larger, 11–15 × 6–9 μm, thallus Pd–	**15**
15	Thallus not shining, apothecia 0.7–4 mm diam., disc not shining, hymenium 80–110 μm high, ascospores 11–15 × 6–9 μm	***L. decorata***
–	Thallus somewhat shining and waxy, apothecia ca 0.5 mm diam., disc shining, hymenium 55–65 μm high, ascospores 11–13 × 6–7 μm	***L. marginata***
16	Disc pale to green–brown or black–green	**17**
–	Disc pale brown, red–brown to dark brown	**19**
17	Thallus somewhat glossy, prothallus whitish to whitish gray when present, apothecia 0.4–0.7 mm diam., disc waxy, pale to greenish orange, ascospores 8–11.5 × 4–6.5 μm, thallus Pd+ pale orange, containing atranorin and chloratranorin	***L. plumosa***
–	Thallus not glossy, prothallus black when present, apothecia 0.3–1 mm diam., disc not waxy, pale yellow to greenish brown or greenish black, ascospores 10–14 × 5–7 μm, thallus Pd–, containing usnic acid and zeorin	**18**
18	Thallus continuous and well-developed, disc green–brown to green–black, epihymenium green–brown to brown, hymenium 60–70 μm high, pycnoconidia 23–25 × 0.5–1 μm, thallus UV+ dull orange	***L. intricata***
–	Thallus inconspicuous with dispersed areoles, disc pale yellow to pale brown, epihymenium hyaline to yellow– or red–brown, hymenium 45–60 μm high, pycnoconidia 18–22 ×1 μm, thallus UV–, containing rangiformic acid and ±eulecanoral	***L. polytropa***
19	Thallus richly sorediate, disc dark brown and shiny	***L. ussuriensis***
–	Thallus not sorediate, disc not shiny	**20**
20	Thallus pale to medium yellow or yellow–green, not white or gray	***L. frustulosa***
–	Thallus pale to white, gray or dark gray	**21**
21	Apothecia smaller, up to ca 0.5 mm diam.	**22**
–	Apothecia larger, 0.5–2.5 mm diam.	**23**
22	Thallus medium to dark gray, epihymenium yellow to brown, ascospores 9–15 × 4–6 μm, often guttulate and appearing 1-septate, reaction all negative else epihymenium K+ yellow	***L. helicopis***
–	Thallus grayish white, epihymenium brownish red, ascospores 12–13 × 5–6 μm, thallus K+ pale yellow, epihymenium K–	***L. subimmersa***
23	Prothallus whitish	**24**
–	Prothallus absent	**28**
24	Thallus pale, gray to dark gray, thalline margin with irregular or large crystals	**25**
–	Thallus white, grayish white or yellowish white, thalline margin with small crystals	**27**
25	Ascospores narrower, 10–15 × 5–7 μm, thallus Pd–	***L. subimmergens***
–	Ascospores wider, 9–15 × 6–8.5 μm, thallus Pd+ weakly yellow or yellow turning to red	**26**
26	Epihymenium pale orange to red–brown without granules, paraphyses tips red–brown, asci 50–60 × 12–21 μm, containing zeorin	***L. campestris***
–	Epihymenium brown to olivaceous brown with coarse granules dissolving in K, paraphyses tips olivaceous, asci 45–50 × 7–9 μm, containing ±roccellic acid and ±norstictic acid	***L. cenisia***
27	Disc orange, red–orange to red–brown, thalline margin with small irregular crystals not dissolving in K, hypothecium without oil droplets, thallus Pd–, containing ±chloratranorin	***L. horiza***
–	Disc brown to dark brown, thalline margin with small and large crystals dissolving in K, hypothecium inspersed with oil droplets, thallus Pd+ light orange, containing zeorin	***L. melacarpella***
28	Ascospores smaller, 8–12 × 4.5–5 μm	***L. orientalis***
–	Ascospores larger, 9–15 × 5–8.5 μm	**29**
29	Thalline margin with only large crystals not dissolving in K, thallus yellowish gray to whitish gray, ascospores narrower, 9–15 × 5–7 μm, prothallus absent	***L. pseudistera***
–	Thalline margin with small crystals, thallus whitish to grayish white, ascospores wider, 10.5–15 × 6.5–8.5 μm, prothallus white when present	**30**
30	Disc orange, red–orange or red–brown, thalline margin with small irregular crystals not dissolving in K, ascospores 12–15 × 6.5–8.5 μm, thallus K+ indistinct yellow, Pd–, containing ±chloratranorin	***L. horiza***
–	Disc brown to dark brown, thalline margin with small and large crystals dissolving in K, ascospores 10.5–13.5 × 7.5–8.5 μm, thallus K+ yellow, Pd+ light orange, containing zeorin	***L. melacarpella***
31	Thallus sorediate	**32**
–	Thallus not sorediate	**39**
32	Apothecia absent or rarely seen	**33**
–	Apothecia present	**35**
33	Thallus UV–, apothecia not seen, containing stictic acid	***L. layana***
–	Thallus UV+ pale orange or ice blue, apothecia rarely seen	**34**
34	Thallus pale gray, prothallus pale gray, apothecia not pruinose, ascospores 7–10 × 3–4 μm, thallus Pd± yellow, UV+ pale orange, containing chloratranorin	***L. barkmaniana***
–	Thallus yellow to greenish, occasionally with blue or gray tints, prothallus white and fibrous, often with one or two blue–gray zones, apothecia faintly or heavily white pruinose, ascospores 11–14 × 6–9 μm, thallus Pd–, UV+ ice blue (or violet), containing usnic acid and porphyrilic acid	***L. thysanophora***
35	Apothecia pruinose	**36**
–	Apothecia not pruinose	**38**
36	Ascospores smaller, 11–14 × 6–9 μm	***L. thysanophora***
–	Ascospores larger, 15–24 × 7–12 μm	**37**
37	Thallus white or yellowish white, asci 8- or 6-spored, ascospores 15–22 × 7–12 μm	***L. pachycheila***
–	Thallus yellowish–blue green, asci 8-spored, ascospores 15–20 × 9–12 μm	***L. sibirica***
38	Thallus pale gray, apothecia 0.4–0.7 mm diam., ascospores 7–10 × 3–4 μm	***L. barkmaniana***
–	Thallus yellowish gray to greenish gray, apothecia 0.2–0.6 mm diam., ascospores 12–15.5 × 6–8.5 μm, containing chodatin, demethlchodatin, and thiophanic acid	***L. leproplaca***
39	Prothallus distinct, whitish, grayish or blackish	**40**
–	Prothallus indistinct or absent	**47**
40	Prothallus whitish or grayish, but not blackish	**41**
–	Prothallus blackish	**44**
41	Thalline margin with large crystals (> 10 μm diam.) not dissolving in K	**42**
–	Thalline margin with small crystals (< 10 μm diam.)	**43**
42	Apothecia pale orange to yellowish brown, sometimes slightly pruinose on disc, epihymenium hyaline to yellow–brown with numerous small crystals (*chlarotera*-type), ascospores 9–13 × 5–7 μm, containing gangaleoidin, chloratranorin, chlorolecideoidin, leoidin, and norgangaleoidin	***L. leprosa***
–	Apothecia dark reddish brown to brownish black, not pruinose, epihymenium reddish brown with fine brown granules (*pulicaris*-type), ascospores 11–14 × 6–8 μm, containing fumarprotocetraric acid, ±roccellic acid	***L. pulicaris***
43	Subhymenium 60–80 μm high, asci consistently 8-spored, ascospores 15–20 × 8–11 μm, pycnidia brown–black with pycnoconidia 20–25 × 0.5 μm, containing zeorin	***L. chionocarpa***
–	Subhymenium 180–220 μm high, asci 8-spored or 16-spored, ascospores 12–16 × 6–8 μm, pycnidia absent	***L. japonica***
44	Thalline margin with granular crystals dissolving in K	***L. megalocheila***
–	Thalline margin with large crystals not dissolving in K	**45**
45	Disc paler, orange–brown or pale red–brown, flat to slightly convex, epihymenium inspersed with coarse granules, thallus Pd–, containing pannarin, ±placodialic acid, and ±roccellic acid	***L. cinereofusca***
–	Disc darker, dark reddish brown to brownish black, flat to slightly concave, epihymenium without granules, thallus Pd+ faintly yellow	**46**
46	Paraphyses tips reddish brown (or faintly yellow), asci wider, 45–55 × 18–22 μm, ascospores larger, 11.5–14.5 × 6–8.5 μm, containing gangaleoidin and usually traces of californin	***L. argentata***
–	Paraphyses tips dark brown, asci narrower, 50–60 × 8–12 μm, ascospores smaller, 9–14 × 5–8 μm, containing zeorin	***L. iseana***
47	Thalline margin finally excluded	**48**
–	Thalline margin permanent	**51**
48	Disc pruinose, thallus not corticate, containing decarboxysquamatic acid	***L. strobilina***
–	Disc not pruinose, thallus corticate, decarboxysquamatic acid absent	**49**
49	Asci 16- or 32-spored, thallus C–, K–, KC–, containing no substance	***L. sambuci***
–	Asci 8-spored, thallus C± orange, KC± yellow to orange, containing usnic acid, zeorin and xanthones	**50**
50	Thallus yellow–green to gray–green, disc pale yellow to greenish, when young the exciple crenulate and containing algae	***L. confusa***
–	Thallus yellowish–white to greenish black, disc pinkish brown to greenish black, when young the exciple smooth and lacking algae	***L. symmicta***
51	Disc and epihymenium darker, brown, red–brown to dark brown, not pruinose	**52**
–	Disc or epihymenium paler, pinkish, pale orangish, green–brown, yellow–brown, orange–brown to pale red–brown, pruinose or not	**57**
52	Thalline margin with small crystals (*allophana*-type) not dissolving in K	**53**
–	Thalline margin with large crystals (*pulicaris*-type)	**54**
53	Thallus darker, bluish, olivish or pale brownish gray, disc brownish, amphithecial cortex present, epihymenium with granules on the surface not dissolving in K, hypothecium shorter, 15–25 μm high, oil droplets present in apothecial section	***L. baekdudaeganensis***
–	Thallus paler, greenish or yellowish gray, disc reddish brown, amphithecial cortex indistinct or absent, epihymenium without granules, hypothecium taller, 50–75 μm high, oil droplets absent	***L. imshaugii***
54	Epihymenium without granules, ascospores 10.5–16.5 × 6–9.5 μm	**55**
–	Epihymenium with coarse, hyaline to brown granules (*chlarotera*-type), ascospores 8–12 × 4.5–7 μm	**56**
55	Apothecia smaller, 0.4–0.8 mm diam., ascospores 11.5–14.5 × 6–8.5 μm, thallus Pd+ weakly yellow, containing gangaleoidin	***L. argentata***
–	Apothecia larger, up to 1.6 mm diam., ascospores 10.5–16.5 × 6–9.5 μm, thallus Pd–, containing zeorin	***L. perplexa***
56	Apothecia not constricted at base, disc slightly convex when mature, hymenium including subhymenium 80–100 μm high, hypothecium 120–150 μm high, asci 40–50 × 7–12 μm, ascospores 8–10 × 4.5–6 μm, pycnidia absent, thallus Pd+ orange	***L. nipponica***
–	Apothecia slightly constricted at base, disc extremely convex when mature, hymenium including subhymenium 150–200 μm high, hypothecium 70–110 μm high, asci 50–60 × 10–15 μm, ascospores 9–12 × 6–7 μm, pycnidia present, thallus Pd–	***L. sulcata***
57	Asci 12- or 16-spored	***L. loekoesii***
–	Asci 8-spored	**58**
58	Ascospores smaller, 7–10 × 4–5 μm, thallus C–, K–, KC– (or KC+ yellow), Pd–	***L. saligna***
–	Ascospores larger, 9–15 × 5–9.5 μm, thallus K+ yellow, Pd+ yellow–orange to orange	**59**
59	Ascospores 9–11.5 × 5–7 μm, thallus K+ weakly yellow, Pd+ sulphur yellow, thallus areoles somewhat shiny, containing psoromic acid and usnic acid	***L. varia***
–	Ascospores 9–15 × 5–9.5 μm, thallus K+ distinct yellow or yellow turning to red, Pd+ pale yellow to yellow orange, thallus not shiny	**60**
60	Thalline margin with numerous small crystals (*allophana*-type), containing ±stictic acid	**61**
–	Thalline margin with large crystals not dissolving in K (*pulicaris*-type)	**62**
61	Disc carneous to pinkish, flat to convex, heavily pruinose, epihymenium gray–brown, thallus K+ yellow or yellow turning to red, Pd+ orange, containing chloratranorin, ±norstictic acid, ±protocetraric acid, ±virensic acid, ±connorstictic acid, ±conprotocetraric acid, and ±salazinic acid	***L. caesiorubella***
–	Disc yellowish brown to dark reddish brown, flat to slightly concave, slightly pruinose, epihymenium dark brown to blackish brown, thallus K+ yellow, Pd+ pale yellow, containing hafellic acid, zeorin, and usnic acid	***L. hafelliana***
62	Thallus pale to medium gray, apothecia larger, 0.7–1.5 mm diam., disc not pruinose, epihymenium red–brown, paraphyses anastomosed, ascospores larger, 10–14.5 × 7–8.5 μm, containing pannarin and ±placodialic acid	***L. cinereofusca***
–	Thallus yellow–white to yellow gray, apothecia smaller, 0.2–1 mm diam., disc slightly pruinose, epihymenium pale to red–brown, paraphyses sparsely branched, ascospores smaller, 9–13.5 × 5–7 μm	**63**
63	Apothecia 0.5–1 mm diam., prothallus absent, thallus Pd–, containing ±roccellic acid and ±fatty acid	***L. hybocarpa***
–	Apothecia 0.2–0.7 mm diam., prothallus gray when present, thallus Pd+ pale orange, containing gangaleoidin, chloratranorin, chlorolecideoidin, leoidin, and norgangaleoidin	***L. leprosa***

## Supplementary Material

XML Treatment for Lecanora baekdudaeganensis

## References

[B1] Arup U Søchting U Frödén P (2013) A new taxonomy of the family Teloschistaceae. Nordic Journal of Botany 31(1): 016–083. 10.1111/j.1756-1051.2013.00062.x

[B2] Bouckaert RR Drummond AJ (2017) bModelTest: Bayesian phylogenetic site model averaging and model comparison. BMC evolutionary biology 17(1): 42 10.1186/s12862-017-0890-6 28166715 PMC5294809

[B3] Brodo IM (1984) The North American species of the *Lecanora subfusca* group. Beihefte zur Nova Hedwigia 79: 63–185.

[B4] Culberson CF (1972) Improved conditions and new data for identification of lichen products by standardized thin-layer chromatographic method. Journal of Chromatography A 72(1): 113–125. 10.1016/0021-9673(72)80013-X 5072880

[B5] Drummond AJ Rambaut A (2007) BEAST: Bayesian evolutionary analysis by sampling trees. BMC evolutionary biology 7(1): 214 10.1186/1471-2148-7-214 17996036 PMC2247476

[B6] Drummond AJ Suchard MA Xie D Rambaut A (2012) Bayesian phylogenetics with BEAUti and the BEAST 1.7. Molecular biology and evolution 29(8): 1969–1973. 10.1093/molbev/mss075 22367748 PMC3408070

[B7] Edler D Klein J Antonelli A Silvestro D (2019) raxmlGUI 2.0 beta: a graphical interface and toolkit for phylogenetic analyses using RAxML. bioRxiv. 10.1101/800912

[B8] Ekman (2001) Molecular phylogeny of the Bacidiaceae (Lecanorales, lichenized Ascomycota). Mycological Research 105: 783–97. 10.1017/S0953756201004269

[B9] Guderley R Lumbsch HT (1999) Notes on multispored species of *Lecanora* sensu stricto. The Lichenologist 31(2): 197–203. 10.1006/lich.1998.0190

[B10] Hall TA (1999) BioEdit: A User-Friendly Biological Sequence Alignment Editor and Analysis Program for Windows 95/98/NT. Nucleic Acids Symposium Series 41: 95–98.

[B11] Hue AM (1915) Lichens novos vel melius cognitos. Annales Mycologici 13: 73–103.

[B12] Kondratyuk SY Lőkös L Halda JP Haji Moniri M Farkas E Park JS Lee BG Oh SO Hur JS (2016) New and noteworthy lichen-forming and lichenicolous fungi 4. Acta Botanica Hungarica 58(1–2): 75–136. 10.1556/034.58.2016.1-2.4

[B13] Korea Forest Service (2019) The Ecological Map of the Baekdudaegan Mountains [Brochure]. Daejeon, South Korea: Korea Forest Service.

[B14] Lendemer JC (2015) *Lecanora layana* (Lecanoraceae), a new sorediate species widespread in temperate eastern North America. The Bryologist 145–153. 10.1639/0007-2745-118.2.145

[B15] Lumbsch HT (1995) A new species in the *Lecanora subfusca* group containing usnic acid in addition to atranorin. The Lichenologist 27(3): 161–167. 10.1016/S0024-2829(95)80015-8

[B16] Lumbsch HT Messuti MI Nash TH (2003) New or Overlooked Species in the *Lecanora subfusca* Group from Southwestern North America (Lecanorales, Ascomycotina). The Bryologist 106(4): 552–559. 10.1639/0007-2745(2003)106[552:NOOSIT]2.0.CO;2

[B17] Lü L Joshi Y Lumbsch HT Wang HY Koh YJ Hur JS (2011) New and noteworthy species of the lichen genus Lecanora (Ascomycota; Lecanoraceae) from South Korea. The Lichenologist 43(4): 321–329. 10.1017/S0024282911000144

[B18] Malíček J (2014) A revision of the epiphytic species of the *Lecanora subfusca* group (Lecanoraceae, Ascomycota) in the Czech Republic. The Lichenologist 46(4): 489–513. 10.1017/S0024282914000139

[B19] Miyawaki H (1988) Studies on the *Lecanora subfusca* group in Japan. Journal of the Hattori Botanical Laboratory 64: 271–326.

[B20] Nylander W (1891) Sertum lichenaeæ tropicæ e Labaun et Singapore. e typis P. Schmidt.

[B21] Orange A James PW White FJ (2001) Microchemical Methods for the Identification of Lichens. The British Lichen Society, London, UK.

[B22] Rambaut A (2014) FigTree v1.4.2. Edinburgh: University of Edinburgh. http://tree.bio.ed.ac.uk/software/figtree

[B23] Schwarz G (1978) Estimating the dimension of a model. Annals of Statistics 6: 461–464. 10.1214/aos/1176344136

[B24] Smith CW Aptroot A Coppins BJ Fletcher A Gilbert OL James PW Wolseley PA (2009) The lichens of Great Britain and Ireland. The British Lichen Society, London, UK.

[B25] Stecher G Tamura K Kumar S (2020) Molecular Evolutionary Genetics Analysis (MEGA) for macOS, Molecular Biology and Evolution 37(4): 1237–1239. 10.1093/molbev/msz312 PMC7086165 31904846

[B26] White TJ Bruns T Lee S Taylor JW (1990) Amplification and direct sequencing of fungal ribosomal RNA genes for phylogenetics. PCR protocols: a guide to methods and applications 18(1): 315–322. 10.1016/B978-0-12-372180-8.50042-1

[B27] Zoller S Scheidegger C Sperisen C (1999) PCR primers for the amplification of mitochondrial small subunit ribosomal DNA of lichen-forming ascomycetes. The Lichenologist 31(5): 511–516. 10.1006/lich.1999.0220

